# Predicting visual field global and local parameters from OCT measurements using explainable machine learning

**DOI:** 10.1038/s41598-025-89557-1

**Published:** 2025-02-16

**Authors:** Md Mahmudul Hasan, Jack Phu, Henrietta Wang, Arcot Sowmya, Erik Meijering, Michael Kalloniatis

**Affiliations:** 1 School of Computer Science and Engineering, University of New South Wales, Sydney, NSW 2052 Australia; 2 School of Optometry and Vision Science, University of New South Wales, Sydney, NSW Australia; 3 Centre for Eye Health, University of New South Wales, Sydney, NSW Australia; 4 Faculty of Medicine and Health, University of Sydney, Camperdown, NSW Australia; 5 School of Medicine (Optometry), Deakin University, Waurn Ponds, VIC Australia; 6 University of Houston College of Optometry, University of Houston, Houston, TX USA

**Keywords:** 24-2 test grid, Optical coherence tomography, Glaucoma, Explainable machine learning, SHAP analysis, Perimetry, Visual fields, Optic nerve diseases, Biomedical engineering

## Abstract

Glaucoma is characterised by progressive vision loss due to retinal ganglion cell deterioration, leading to gradual visual field (VF) impairment. The standard VF test may be impractical in some cases, where optical coherence tomography (OCT) can offer predictive insights into VF for multimodal diagnoses. However, predicting VF measures from OCT data remains challenging. To address this, five regression models were developed to predict VF measures from OCT, Shapley Additive exPlanations (SHAP) analysis was performed for interpretability, and a clinical software tool called OCT to VF Predictor was developed. To evaluate the models, a total of 268 glaucomatous eyes (86 early, 72 moderate, 110 advanced) and 226 normal eyes were included. The machine learning models outperformed recent OCT-based VF prediction deep learning studies, with correlation coefficients of 0.76, 0.80 and 0.76 for mean deviation, visual field index and pattern standard deviation, respectively. Introducing the pointwise normalisation and step-size concept, a mean absolute error of 2.51 dB was obtained in pointwise sensitivity prediction, and the grayscale prediction model yielded a mean structural similarity index of 77%. The SHAP-based analysis provided critical insights into the most relevant features for glaucoma diagnosis, showing promise in assisting eye care practitioners through an explainable AI tool.

## Introduction

Glaucoma, a leading cause of blindness worldwide^1^, manifests as progressive and irreversible vision loss, significantly impacting patients’ quality of life^2^. The disease is characterised by gradual damage to the visual field (VF)^3–5^, due to structural changes such as ganglion cell (GC) death and loss of axons^6,7^, resulting in deficits in the retinal nerve fibre layer (RNFL) and thinning of the neuroretinal rim^8^. Spectral-domain optical coherence tomography (OCT) accurately evaluates these alterations by measuring peripapillary RNFL, GC inner plexiform layers (IPL), and macular (MC) thickness^9^. Patients suspected of suffering from glaucoma typically undergo static automated perimetry (SAP)^10^ that may use the Humphrey 24-2 test grid Swedish Interactive Threshold Algorithm (SITA)^4,5^ (HVF 24-2), the most widely used method for VF testing^11^.

Performing visual field (VF) tests, particularly using SAP, the most common method for assessing VF, can be quite challenging in some patient populations, potentially compromising the reliability and accuracy of the results. Elderly patients, particularly those with decreased attention spans or poor eye-hand coordination due to physiological ageing or systemic comorbidities, often struggle with VF testing^12^. Additionally, patients with severe physical limitations, neck problems, or those who experience significant discomfort during testing may refuse to participate or may provide unreliable results^12^. Especially, patients with neurological conditions such as Parkinson’s disease^13^ or Alzheimer’s disease^14^ may have difficulty following instructions or pressing the response button consistently. Those taking medications that cause drowsiness or affect the central nervous system can also experience reduced performance during VF tests and non-cooperative patients may be unable to complete the test altogether^15^. Beyond these patient-specific challenges, SAP itself has inherent limitations including subjectivity, high intra-subject variability, lengthy testing time, and the need for specific testing facilities^16^. Moreover, once moderate VF loss occurs, test–retest variability increases substantially^17,18^, limiting reliable assessment of change. In such cases, clinicians may need to rely more heavily on other diagnostic tools like OCT scans or clinical examination findings to monitor glaucoma progression, despite the challenges of depending solely on structural imaging for decision-making.

OCT is quick and relatively easy to capture from the patient’s eye, providing detailed and non-invasive images of the retinal layers^19^. Its advantages include high resolution, the ability to detect early structural changes, and the convenience of integration into routine clinical practice^20^. If OCT is available, it can offer predictive insights into VF sensitivity, helping guide clinicians toward possible multimodal diagnoses and informed management strategies. Despite the known structure–function relationship, accurately predicting VF sensitivity from OCT-measured retinal layer thickness remains elusive^21^, leading to separate interpretations of spectral domain (SD)-OCT and VF results in clinical settings. Recent innovations in artificial intelligence (AI) based models show promise in predicting functional outcomes (VF) from structural OCT measurements.

Recent structure-functional cross-modal translation studies are mostly focused on using deep learning, more specifically deep convolutional neural networks (CNNs), which use structural data as input and generate VF pointwise threshold sensitivity (TS) values and mean deviation (MD). For example, Mohammadzadeh et al.^22^ used OCT 3D volume scans from Spectralis OCT as input to 3D-DenseNet121 and obtained a Pearson correlation (R) of 0.74 and mean absolute error (MAE) of 3.5 dB in MD prediction and MAE of 6.5 dB in TS prediction of 10–2 VF (68 locations). Mark et al.^23^ utilised spectral domain OCT (SD-OCT) optic nerve head (ONH) images and used ResNet50 and obtained a MAE of 3.7 dB in MD prediction. They have reported an intriguing observation that when predicting functional outcomes in the inferior and inferior nasal VF sector pointwise TS, the models predominantly relied on structural information from the superior ONH region. Similarly, the inferior ONH regions were utilised by deep learning models to predict functional outcomes in the superior and superior nasal VF sectors. Hemelings et al.^24^ used a customised deep learning regression model with Xception backbone applied to circumpapillary OCT scans and achieved a MAE of 2.89 dB (95% CI 2.50–3.30 dB) in MD prediction and 4.82 dB (95% CI 4.45–5.22 dB) in TS prediction. Zhu et al.^25^ utilised RNFL thickness measurements from scanning laser polarimetry (SLP) and obtained MAE of 4.9 dB (standard deviation: 4.0) using a radial basis function customised under a Bayesian framework (BRBF) while predicting TS values. Using SD-OCT scans, Lazaridis et al.^26^ obtained a MAE of 3.6 dB with a multichannel variational autoencoder (MC-VAE). Similarly, Moon et al.^27^ obtained a MAE of 3.10–3.17 dB using Inception-ResNet-V2 applied to swept-source optical coherence tomography (SS-OCT) images.

From this review of the previous structure–function studies, it can be found that most of them have used raw OCT images as input and trained deep learning networks for VF prediction. However, deep learning models are computationally expensive^28^ and typically lack explanation and reasoning for their predictions^29^. To develop a trustworthy and responsible AI-based system, explainability is critical^29,30^. On the other hand, the built-in software of commercial OCT machines, such as CIRRUS HD OCT (Zeiss, Germany), provides interpretable analyses using both eyes (oculus uterque (OU) analysis), such as RNFL thickness analysis, GC-IPL thickness analysis and MC thickness analysis, and it is possible to use these extracted features combinedly to predict VF from OCT using ML. Moreover, when previous studies predicted VF, they mostly predicted pointwise threshold sensitivity (TS) values, with some including the blind spots^21,27,31^, which may be prone to noise. A small number of studies predicted the grayscale image, which might add utility to clinicians.

Considering the above research gaps and limitations, this cross-sectional study aims to predict VF local (52 data points in total and grayscale image) and global indices, i.e., MD, pattern standard deviation (PSD) and visual field index (VFI), using ML from OCT-derived parameters of RNFL, GC and MC thickness. We also use an explainable machine learning (XML) technique, namely SHapley Additive exPlanations (SHAP) analysis, to evaluate our prediction model with respect to the MD prediction, which is novel and not attempted before for this problem. Overall, our study assesses the trustworthiness of an OCT-to-VF prediction tool, with a focus on explainability to build a reliable and user-friendly software tool that can aid clinicians in diagnosing glaucoma.

## Materials and methods

### Data acquisition and labelling

Ethics approval was obtained from the University of New South Wales (HC210563), and all individuals gave written informed consent allowing the use of their de-identified clinical data for the research. The study followed the principles and regulations of the Declaration of Helsinki. Data was acquired from patients who attended the Centre for Eye Health (CFEH) from 2015 to 2021. The diagnosis of glaucoma and other ocular conditions followed CFEH procedures and protocols^32–35^, involving a thorough review of clinical data by a senior clinician, optometrist, or ophthalmologist. Furthermore, an additional expert conducted supplementary examinations to facilitate inclusion in the study. CFEH is a referral-only clinic and diagnosis and clinical audits also exist within the centre^33^. To ensure accurate measurements from OCT images, rigorous image quality control criteria were applied. As in previous studies^4,36^, OCT images from the CIRRUS HD OCT machine were included only if they met the following standards: a signal strength of ≥ 6 (on a scale of 1 to 10), absence of significant motion artefacts, proper centration of the scan on the optic nerve head or macula, and no segmentation errors determined by visual inspection by an experienced clinician. Images failing to meet these criteria were excluded from further analysis. In this study, glaucoma diagnoses were classified into three categories based on MD of VFs (early glaucoma, moderate glaucoma, and advanced glaucoma) using criteria by Mills et al.^37^, with the advanced stage defined by MD, while a central field defect^38^ was not considered advanced unless it met the MD criterion. Healthy individuals (n = 226 eyes) were age similar to glaucoma patients (n = 268 eyes) for reference labels in training a machine learning model (Table 1).

**Table 1:** Characteristics of the participants involved in this research study.

Glaucoma types	N	Age (mean ± std)	RE	LE	RE + LE
Normal	113	68.68 ± 6.81	114	112	226
Early	86	66.34 ± 8.86	48	38	86
Moderate	72	65.90 ± 11.11	36	36	72
Advanced MD	37	64.70 ± 10.83	24	13	37
Advanced (central VF Defect)	73	61.78 ± 13.39	39	34	73
Glaucomatous optic neuropathy (total)	268	64.48 ± 11.44	147	121	268

*RE* Right eye, *LE* Left eye.

### Feature extraction, imputation and regression analysis

A total of 45 spatial domain features such as RNFL, GC-IPL, and MC thickness were extracted from OCT images to analyse the retinal layer distribution and variations (Supplementary Fig. 1, Supplementary Table 1), which have been found clinically useful to differentiate between glaucoma, glaucoma suspects and healthy groups^39^. The CIRRUS HD OCT software facilitated this extraction, generating numerical data for further analysis. For patients with data artifacts, multiple imputation using chained equations (MICE)^40^ was employed to impute partially missing data (5.22% of total samples) based on existing artifact-free parameters. Artifacts included issues like image truncation, inaccurate cup or disc margin delineation and media opacities affecting RNFL, GC-IPL, or MC measurements. Patients with at least one artifact-free parameter (e.g., RNFL) were included, and artifact-affected parameters (e.g., GC-IPL) were treated as missing data. MICE imputation used the existing artifact-free data (e.g., RNFL and MC) to reconstruct these values. However, when all the RNFL, GC-IPL or Macular thickness data were affected by artefacts, the sample was removed from the dataset.

**Fig. 1:**
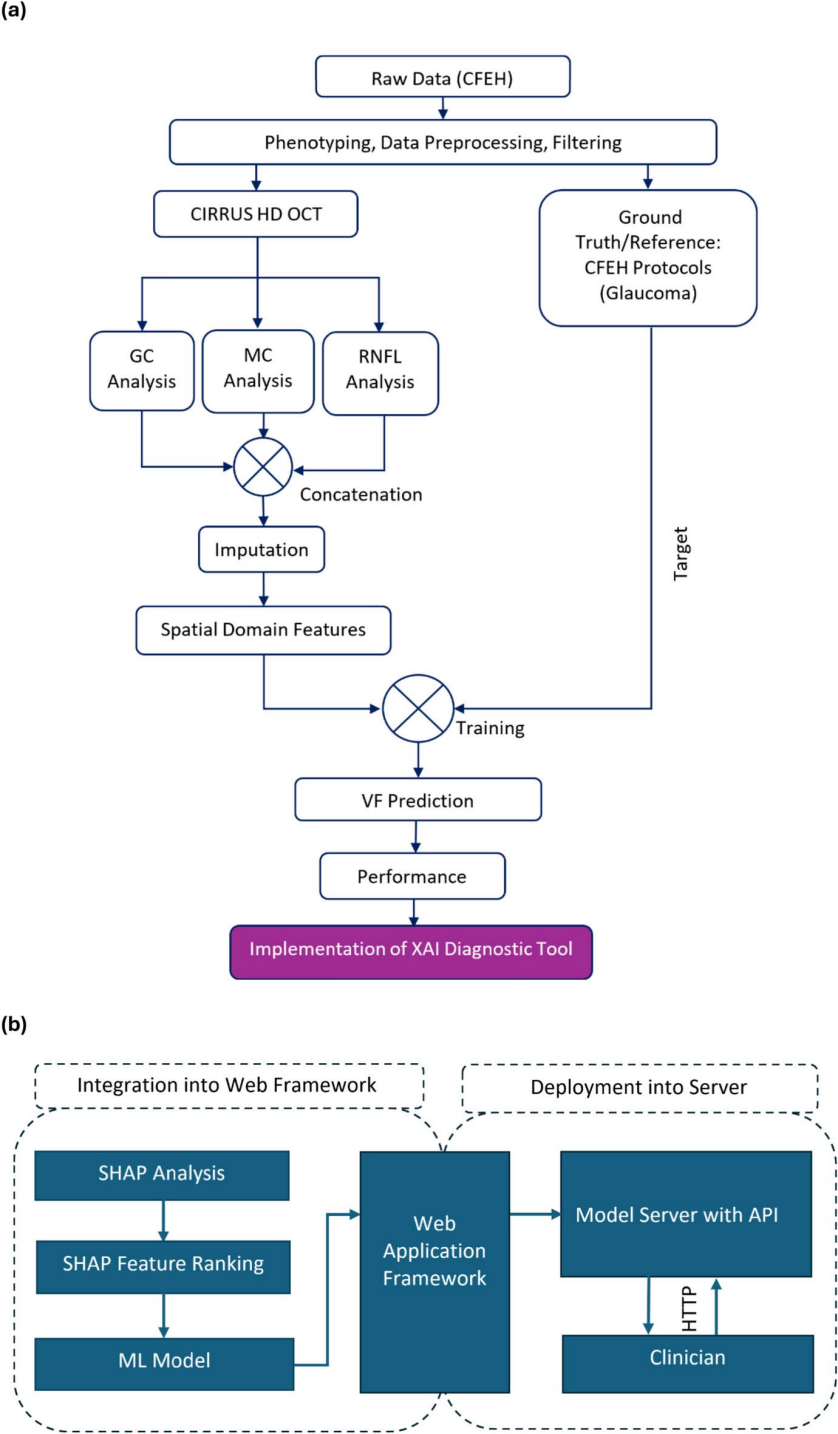
(**a**) Flowchart of the proposed methodology of this study involves several key stages, including the phenotyping of raw data, extraction of features in the spatial domain, selection of relevant features, VF prediction, and model interpretation employing eXplainable Machine Learning (XML). (**b**) Flowchart for implementing the OCT to VF prediction tool (glaucoma vs non-glaucoma) based on explainable machine learning. Blue-coloured blocks represent explainable analysis.

The 24-2 VF data (pointwise sensitivity and global indices MD, VFI and PSD) was extracted using a custom MATLAB program (MathWorks, Natick, MA) which uses optical character recognition, converting image to text^41^. All the extracted sensitivities and indices were used as target outputs for the regression model with OCT inputs. Based on distinct algorithms^42,43^, three supervised regression models, namely XGBoost, a Support Vector Machine (SVM), and a Random Forests (RF) regressor were trained for predicting VF from OCT, with hyperparameters fine-tuned through iterative processes using Gridsearch algorithm for optimal accuracy. Feature scaling was applied to SVM^44^, while XGBoost and RF did not require it due to algorithm robustness, aiding the explanation of features using explainable machine learning (XML). Data imbalance was addressed using the synthetic minority over-sampling technique (SMOTE)^45^, which augmented the minority group (normal subjects) to have the same number of samples as the glaucoma patients. All models were implemented in Python 3.7 on Google Colab platform^46^. Although we applied MICE for imputing missing OCT features and SMOTE for oversampling (normal: diseased imbalance ratio: 0.84), we ran another sub-analysis with the original (clean) data, excluding any subject having missing values. Again, another sub-analysis was performed by training the models with the clean data and testing with the augmented data to assess the robustness of the models to the augmented dataset. After initial model training, SHAP analysis was applied to the original dataset (without augmentation) to interpret the regression models, and a selected feature set was used to develop the final prediction tool (Fig. 1).

### Performance measures

ML regression performance was assessed using various metrics: mean absolute error (MAE), root mean square error (RMSE), correlation coefficient (R) and mean structural similarity index (MSSI)^47,48^. MAE measures the average absolute difference between predicted and actual values while RMSE emphasises larger errors by computing the square root of the average squared differences:

1 $$\text{MAE}=\frac{1}{n}{\sum }_{i=1}^{n}\left|{y}_{i}-{\widehat{y}}_{i}\right|$$

2 $$\text{RMSE}=\sqrt{\frac{1}{n}{\sum }_{i=1}^{n}{({\widehat{y}}_{i}- {y}_{i})}^{2}}$$

Here, $n$ represents the total number of samples, ${y}_{i}$ denotes the actual value of the *i*-th sample, and $\widehat{y}$ represents the predicted value of the *i*-th sample. Lower values of MAE and RMSE indicate better performance.

The Pearson correlation coefficient indicates the linear relationship between predicted values (by the regression model) and actual values, with values ranging from − 1 to 1.

3 $$R=\frac{{\sum }_{i=1}^{n}({y}_{i}- \overline{y })({\widehat{y}}_{i}- \overline{{\widehat{y} }_{i}})}{\sqrt{{\sum }_{i=1}^{n}{({y}_{i}- \overline{y })}^{2}{\sum }_{i=1}^{n}{({\widehat{y}}_{i}- \overline{{\widehat{y} }_{i}})}^{2}}}$$

Here, $\overline{y }$ is the mean of the actual values, and $\overline{{\widehat{y} }_{i}}$ is the mean of the predicted values. Higher values of R indicate better performance.

The structural similarity index (SSI)^49^ quantifies the similarity between predicted and actual values, which is important for image analysis tasks. The assessment is done by comparing the similarity between images, considering luminance, contrast, and structure, resulting in a value between -1 and 1, where higher values indicate greater similarity. The SSI is computed over local windows in the images, which allows for capturing both global and local structural information. The inclusion of luminance, contrast, and structure components in the formula makes SSI a useful metric for evaluating the perceptual quality of images, which is particularly important in applications like image compression, image restoration, and image generation. The SSI is calculated as^47^:

4 $$\text{SSI} (x,y)=\frac{(2{\mu }_{x}{\mu }_{y}+ {c}_{1})(2{\sigma }_{xy}+ {c}_{2})}{({{\mu }_{x}}^{2}+ {{\mu }_{y}}^{2}+ {c}_{1})({{\sigma }_{x}}^{2}+ {{\sigma }_{y}}^{2}+ {c}_{2})}$$

where ${\mu }_{x} {\text{ and }}{ \mu }_{y}$ are the mean of the original and predicted images respectively, ${\sigma }_{x} {\text{ and }} {\sigma }_{y}$ are the corresponding standard deviations, and ${c}_{1}$ and ${c}_{2}$ are constants. The similarity index between the original image $x$ and the predicted image $y$ is determined by the combination of three quantities: luminance (L), contrast (C) and structure (S):

5 $$\text{Luminance } \, L(x,y)=\frac{2{\mu }_{x}{\mu }_{x}+ {c}_{1}}{{{\mu }_{x}}^{2}+ {{\mu }_{y}}^{2}+ {c}_{1}}$$

6 $$\text{Contrast} \, C (x,y)=\frac{2{\sigma }_{x}{\sigma }_{y}+ {c}_{2}}{{{\sigma }_{x}}^{2}+ {{\sigma }_{y}}^{2}+ {c}_{2}}$$

7 $$\text{Structure } \, S(x,y)=\frac{{\sigma }_{xy}+ {c}_{3}}{{\sigma }_{x}{\sigma }_{y}+ {c}_{3}}$$

where ${c}_{1}$, ${c}_{2}$ and ${c}_{3}$ are constants. When SSIM is computed in practice, ${c}_{3}$ is often absorbed into ${c}_{2}$ because the structure term is factored into the overall contrast term. To ensure robust and reliable results, it is recommended to train and evaluate models on separate datasets^50,51^. In this study, separate samples for left and right eyes were available for some patients, so patient-level splitting was employed to prevent potential data leakage. Additionally, the robustness of the models was thoroughly evaluated using five-fold cross-validation^50^, with reported results including mean and standard deviations.

### Explainable machine learning (XML)

We have utilised a model-agnostic explainable analysis method named SHAP. It is a method for interpreting ML model predictions by attributing them to the contributing features, utilising Shapley values from game theory to distribute the “credit” fairly^52^. These values assess feature importance and have been applied in various domains, including medicine^53^. Our study employed SHAP-based feature importance, SHAP dependence plot, and SHAP interaction plot to analyse feature contributions^54^. SHAP, a unified framework, extends Shapley values from cooperative game theory, and the combined values are commonly referred to as ‘SHAP values’^54^.

### Prediction tool

Utilising SHAP-based feature importance, we have developed a web application incorporating a ML model. The deployment process involved integrating the model into a web application framework and subsequently executing it onto a server (Fig. 1b). Clinicians can then input necessary features (such as mean RNFL thickness and percentage of RNFL symmetry) and obtain predicted outcomes, such as VF global indices (MD, VFI and PSD values), and local threshold sensitivity values. The tool prioritises the most crucial features identified through SHAP analysis, allowing the model to autonomously determine their utilisation. Validation of the likelihood scores generated by the application involves plotting the predicted probabilities against the MD values of individual samples.

## Results

### Prediction of global indices

#### Mean deviation (MD)

**Fig. 2:**
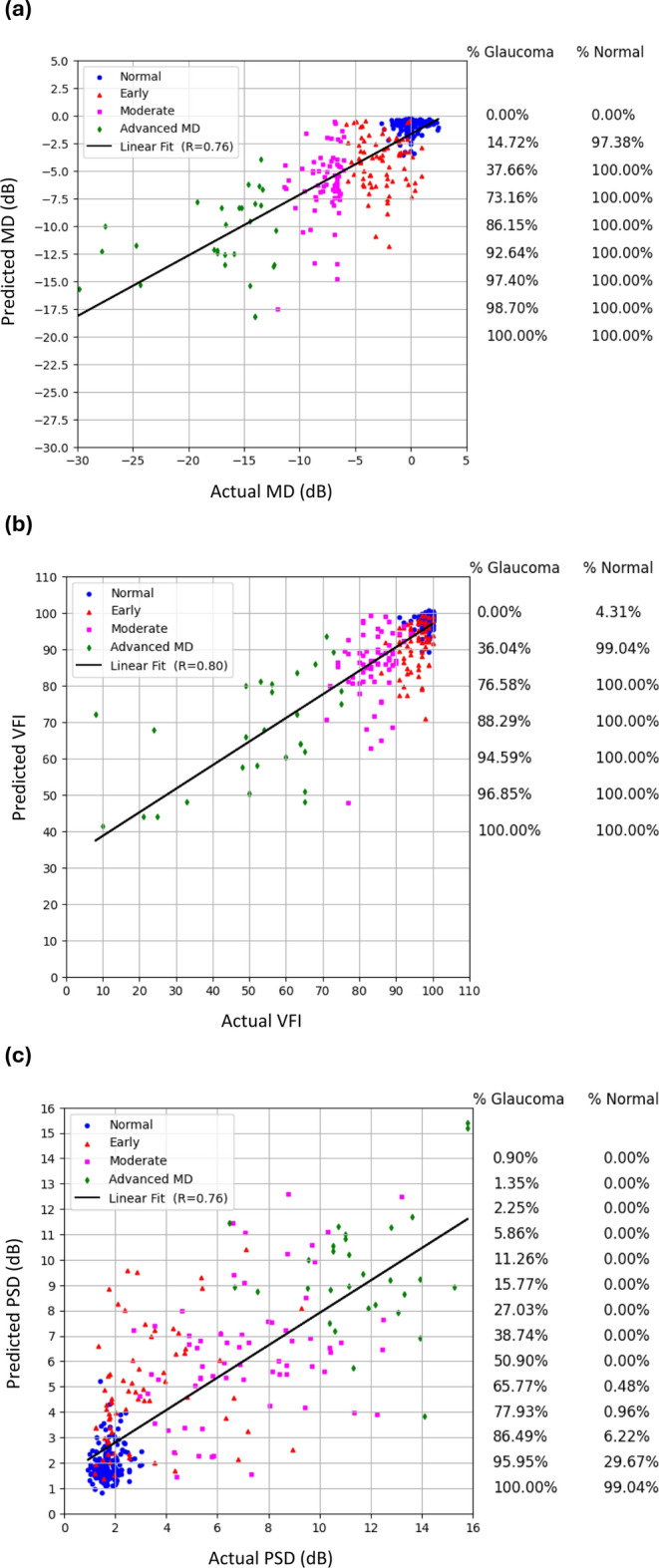
Scatter plots to visualise the correlation between actual and predicted global indices. Among the XGBoost, SVM and RF regressors, the best-performing regressor’s results are presented. (**a**) Mean Deviation (MD), R = 0.76 using a RF regressor. (**b**) Visual Field Index (VFI), R = 0.80 using a XGBoost regressor. (**c**) Pattern Standard Deviation (PSD), R = 0.76 using a XGBoost regressor. The legend on the right side indicates the percentage of glaucoma and normal above the specific thresholds of the global indexes (MD/PSD/VFI).

The prediction of global index MD was performed with three different regressors (XGBoost, SVM and RF), using the RNFL, GC and MC thickness features as input. The performance of the regressors was evaluated using multiple metrics (MAE, RMSE, and R). Using the MICE augmented and SMOTE-oversampled data, all the regressors showed promising performance, with MAE ranging from 2.28 to 2.54 dB and RMSE ranging from 3.30 to 3.64, and RF having the lowest MAE of 2.28 dB and RMSE of 3.30 dB (Table 2). The range of R for all regressors is 0.74–0.76, with XGBoost having a R of 0.74 (95% CI 0.69–0.78), SVM having an R of 0.74 (95% CI 0.69–0.80), and the RF-based model producing the highest R of 0.76 (95% CI 0.70–0.82) (Table 2, Fig. 2). The number of ‘hits’ for correctly predicted glaucoma and normal subjects were also counted for specific MD thresholds, showing that the normal predicted data points are concentrated above a − 2.5 dB threshold, with 97.38% of cases being predicted as normal (Fig. 2a). Conversely, 98.70% of cases were predicted as glaucoma above a − 17.5 dB threshold.

**Table 2:** Performance of the three different regression models for VF prediction from OCT data (including augmented data using MICE and SMOTE) with fivefold cross-validation (report on test results with fivefold cross-validation, mean ± standard deviation).

ML Model	Folds	MD			VFI			PSD		
		R	RMSE (dB)	MAE (dB)	R	RMSE	MAE	R	RMSE (dB)	MAE (dB)
XGBoost	Fold-1	0.80	3.14	2.24	0.89	7.27	4.29	0.85	1.95	1.32
	Fold-2	0.74	3.35	2.30	0.72	9.09	4.67	0.69	2.51	1.70
	Fold-3	0.75	3.50	2.47	0.85	7.72	4.75	0.76	2.48	1.66
	Fold-4	0.73	3.80	2.80	0.75	10.05	4.90	0.77	2.43	1.61
	Fold-5	0.66	4.43	2.91	0.81	7.44	4.79	0.75	2.34	1.47
	**Mean ± STD**	0.74 ± 0.05	3.64 ± 0.5	2.54 ± 0.3	**0.80 ± 0.07**	**8.31 ± 1.21**	**4.68 ± 0.23**	**0.76 ± 0.06**	**2.34 ± 0.23**	**1.55 ± 0.16**
	**95% CI**	0.69–0.78	3.21–4.08	2.28–2.8	0.74–0.86	7.26–9.37	4.47–4.88	0.71–0.81	2.14–2.54	1.42–1.69
SVM	Fold-1	0.79	3.23	1.95	0.75	12.29	5.89	0.76	2.51	1.57
	Fold-2	0.71	3.46	2.38	0.73	9.80	4.88	0.71	2.56	1.63
	Fold-3	0.80	3.26	2.24	0.78	10.66	5.86	0.83	2.51	1.69
	Fold-4	0.77	3.24	2.36	0.68	12.45	6.03	0.75	2.33	1.66
	Fold-5	0.65	4.62	2.92	0.66	10.03	5.47	0.68	2.56	1.62
	**Mean ± STD**	0.74 ± 0.06	3.56 ± 0.6	2.37 ± 0.35	0.72 ± 0.05	11.05 ± 1.25	5.63 ± 0.47	0.75 ± 0.06	2.49 ± 0.10	1.63 ± 0.05
	**95% CI**	0.69–0.80	3.03–4.09	2.06–2.68	0.68–0.76	9.95–12.14	5.22–6.03	0.7–0.79	2.41–2.58	1.59–1.67
RF	Fold-1	0.78	2.80	2.12	0.80	6.73	4.41	0.74	2.76	1.90
	Fold-2	0.75	4.11	2.66	0.80	10.51	5.97	0.75	2.20	1.64
	Fold-3	0.76	3.34	2.31	0.75	8.66	5.23	0.65	2.57	1.81
	Fold-4	0.85	2.67	1.83	0.84	6.86	4.16	0.86	1.92	1.34
	Fold-5	0.67	3.61	2.49	0.66	10.04	6.18	0.70	2.63	1.76
	**Mean ± STD**	**0.76 ± 0.07**	**3.30 ± 0.59**	**2.28 ± 0.32**	0.77 ± 0.07	8.56 ± 1.75	5.19 ± 0.90	0.74 ± 0.08	2.42 ± 0.35	1.69 ± 0.22
	**95% CI**	0.70–0.82	2.78–3.82	2.00–2.56	0.71–0.83	7.03–10.09	4.40–5.98	0.67–0.80	2.11–2.72	1.50–1.88

Boldface numbers indicate the best performance for that metric. Input features included RNFL, GC-IPL and MC thickness data in the spatial and frequency domain. 95% CI = Mean ± 1.96 × Standard Error (SE), SE = STD/$\sqrt{\text{n}}$, n = 5, *CI*:Confidence Interval, *MD* mean deviation, *VFI* visual field index, *PSD* pattern standard deviation, *RMSE* root mean squared error, *MAE* mean absolute error.

Using the original dataset without any augmentation, the regressors showed improved performance compared to the evaluation with augmented data in MD prediction, generating a mean MAE ranging from 2.21 (RF)–2.32 (SVM), RMSE ranging from 3.12 (RF)–3.55 (SVM) and R ranging from 0.73 (SVM)–0.77 (RF) (Table 3). To observe the effect of the augmented data in the prediction, the models were trained using the original clean data without augmentation and tested with only augmented data, which showed a comparatively lower performance, generating a mean MAE ranging from 2.51 (SVM)–3.02 (XGBoost), RMSE ranging from 3.91 (RF)–3.96 (SVM) and R ranging from 0.71 (XGBoost)–0.74 (RF) (Supplementary Table 2). These results show that the model’s performance was reduced while using the partially augmented data as the test set; however, the MAE is within the standard test–retest variability^55^, showing the robustness of the models in prediction.

**Table 3:** Performance of the three different regression models for VF prediction from OCT data (excluding any augmentation using MICE and SMOTE) with fivefold cross-validation (report on test results with fivefold cross-validation, mean ± standard deviation).

ML model	Folds	MD			VFI			PSD		
		R	RMSE (dB)	MAE (dB)	R	RMSE	MAE	R	RMSE (dB)	MAE (dB)
XGBoost	Fold-1	0.78	3.07	1.97	0.79	8.10	4.46	0.76	2.27	1.44
	Fold-2	0.80	2.87	1.99	0.76	7.94	4.91	0.71	2.54	1.83
	Fold-3	0.77	3.76	2.51	0.82	9.15	5.08	0.74	2.61	1.68
	Fold-4	0.63	3.84	2.68	0.68	8.68	5.46	0.65	2.61	1.77
	Fold-5	0.81	2.62	2.00	0.83	6.52	4.61	0.63	2.70	1.81
	**Mean ± STD**	0.76 ± 0.08	3.23 ± 0.54	2.23 ± 0.34	**0.78 ± 0.06**	**8.08 ± 0.99**	**4.91 ± 0.39**	0.70 ± 0.06	2.55 ± 0.16	1.70 ± 0.16
	**95% CI**	0.69–0.82	2.76–3.71	1.93–2.53	0.72–0.83	7.21–8.95	4.56–5.25	0.65–0.75	2.4–2.69	1.56–1.84
SVM	Fold-1	0.75	3.45	2.03	0.69	10.18	5.02	0.72	2.45	1.51
	Fold-2	0.78	3.24	2.21	0.75	9.34	5.45	0.77	2.36	1.70
	Fold-3	0.77	4.23	2.60	0.72	13.37	6.29	0.73	2.76	1.75
	Fold-4	0.64	3.65	2.50	0.62	9.64	5.28	0.7	2.37	1.64
	Fold-5	0.73	3.18	2.26	0.70	9.14	5.08	0.69	2.48	1.70
	**Mean ± STD**	0.73 ± 0.06	3.55 ± 0.42	2.32 ± 0.23	0.70 ± 0.05	10.34 ± 1.74	5.43 ± 0.52	0.72 ± 0.03	2.48 ± 0.16	1.66 ± 0.09
	**95% CI**	0.68–0.78	3.18–3.92	2.12–2.52	0.65–0.74	8.81–11.86	4.97–5.88	0.69–0.75	2.34–2.63	1.58–1.74
RF	Fold-1	0.84	2.73	1.85	0.76	8.29	4.65	0.79	2.12	1.37
	Fold-2	0.78	3.01	2.09	0.78	7.53	4.97	0.79	2.22	1.69
	Fold-3	0.83	3.26	2.29	0.78	10.10	5.45	0.77	2.53	1.57
	Fold-4	0.64	3.77	2.60	0.66	8.96	5.61	0.72	2.33	1.59
	Fold-5	0.78	2.82	2.20	0.81	6.68	4.47	0.71	2.41	1.65
	**Mean ± STD**	**0.77 ± 0.08**	**3.12 ± 0.42**	**2.21 ± 0.28**	0.76 ± 0.06	8.31 ± 1.31	5.03 ± 0.49	**0.75 ± 0.04**	**2.32 ± 0.16**	**1.57 ± 0.12**
	**95% CI**	0.71–0.84	2.75–3.48	1.96–2.45	0.7–0.81	7.16–9.47	4.6–5.46	0.72–0.79	2.18–2.46	1.47–1.68

Boldface numbers indicate the best performance for that metric. Input features included RNFL, GC-IPL and MC thickness data in the spatial and frequency domain. 95% CI = Mean ± 1.96 × Standard Error (SE), SE = STD/$\sqrt{\text{n}}$, n = 5, *CI*: Confidence Interval, *MD* mean deviation, *VFI* visual field index, *PSD* pattern standard deviation, *RMSE* root mean squared error, *MAE* mean absolute error.

#### Visual field index (VFI)

A sub-analysis was performed to predict VFI using the OCT data and the three regressors (XGBoost, SVR and RF) utilising the same feature set that was used for the previous regression analysis (RNFL, GC and MC thickness features). Using the MICE augmented and SMOTE-oversampled data, the regressors showed promising performance (MAE range: 4.68–5.63, mean RMSE range: 8.31–11.05), with SVM having the highest MAE of 5.63 (95% CI 5.22–6.03) and RMSE of 11.05 (95% CI 9.95–12.14), RF having an MAE of 5.19 (95% CI 4.40–5.98) and RMSE of 8.56 (95% CI 7.03–10.09), and the XGBoost model producing the lowest MAE of 4.68 (95% CI 4.47–4.88) and RMSE 8.31 (95% CI 7.26–9.37) (Table 2, Fig. 2). The R range for all regressors was 0.72–0.80, with XGBoost having the highest R of 0.80 (95% CI 0.74–0.86), SVM yielding the lowest R of 0.72 (95% CI 0.68–0.76), and RF producing a R of 0.77 (95% CI 0.71–0.83) (Table 2, Fig. 2). Form the number of ‘hits’ for VFI thresholds, the normal predicted data points are concentrated above the VFI value of 90, with 99.04% normal cases above that threshold, while 94.59% predicted data points were glaucoma above the 60 VFI index (Fig. 2b).

Using the original dataset without any augmentation, the regressors showed somewhat similar performance as the evaluation with augmented data in VFI prediction, generating a mean MAE ranging from 4.91 (XGBoost)–5.43 (SVM), RMSE ranging from 8.08 (XGBoost)–10.34 (SVM) and R ranging from 0.70 (SVM)–0.78 (XGBoost) (Table 3). When the models were trained using the original clean data without augmentation and tested with only augmented data, the models showed a comparatively lower performance, generating a mean MAE ranging from 6.09 (XGBoost)–6.45 (SVM), RMSE ranging from 8.22 (RF)–12.73 (SVM) and R ranging from 0.68 (SVM)–0.78 (RF) (Supplementary Table 2). These results show that the model’s performance was reduced while using the partially augmented data as the test set, but do confirm the robustness of the models in prediction.

#### Pattern standard deviation (PSD)

Another sub-analysis was performed for predicting PSD from the OCT data. Using the MICE augmented and SMOTE-oversampled data, XGBoost achieved the lowest MAE of 1.55 dB (95% CI 1.42–1.69) and RMSE of 2.34 (95% CI 2.14–2.54), SVM achieved a MAE of 1.63 dB (95% CI 1.59–1.67) and RMSE of 2.49 (95% CI 2.41–2.58) and RF achieved a MAE of 1.69 dB (95% CI 1.50–1.88) and RMSE of 2.42 (95% CI 2.11–2.72). The R range for all regressors was 0.74–0.76, with XGBoost having the highest R of 0.76 (95% CI 0.71–0.81), RF having a lower R of 0.75 (95% CI 0.70–0.79), and RF producing the lowest R of 0.74 (95% CI 0.67–0.80) (Table 2, Fig. 2). Form the number of ‘hits’ for PSD thresholds, the predicted data points corresponding to normal subjects are concentrated below the PSD value of 2 dB, with 70.33% normal cases below that threshold, while 86.49% predicted data points were glaucoma above 3 dB of the PSD threshold (Fig. 2c).

Using the original dataset without any augmentation, the regressors showed somewhat similar performance to the evaluation with augmented data in PSD prediction, generating a mean MAE ranging from 1.57 (RF)–1.70 (XGBoost), RMSE ranging from 2.32 (RF)–2.55 (XGBoost) and R ranging from 0.70 (XGBoost)–0.75 (RF) (Table 3). When the models were trained using the original clean data without augmentation and tested with only augmented data, the models showed a comparatively lower performance, generating a mean MAE ranging from 1.16 (RF)–2.21 (SVM), RMSE ranging from 1.65 (RF)–3.17 (SVM) and R ranging from 0.67 (XGBoost)–0.82 (RF) (Supplementary Table 2). These results show that the model’s performance was reduced while using the partially augmented data as the test set, but do confirm the robustness of the models in prediction.

In summary, using the dataset with augmentations, we obtained a higher correlation for predicted global measures: R of 0.76 (RF), 0.80 (XGBoost), and 0.76 (XGBoost) respectively for MD, VFI and PSD. If we consider the other metrics, the lowest MAE is 2.28 dB (RF), 4.67 dB (XGBoost) and 1.55 dB (XGBoost) respectively for MD, VFI and PSD prediction. Using the clean data, we obtained the highest R of 0.77 (RF), 0.78 (XGBoost), and 0.75 (RF), and the lowest MAE is 2.21 dB (RF), 4.91 dB (XGBoost) and 1.57 dB (RF), respectively for MD, VFI and PSD prediction. It demonstrates that using the augmented and clean data, the results are somewhat similar, indicating robustness against OCT artefacts.

The relationship between the other global indices and MD was plotted to observe the correlation between them (Supplementary Fig. 2). The plots show that the actual VFI is positively and highly correlated with actual MD (R = 0.96 using XGBoost), and actual PSD is highly but negatively correlated with MD (R = − 0.83 using XGBoost). When the predicted VFI and PSD were plotted against the actual MD, the correlation coefficients were R = 0.80 and R = − 0.73 respectively for VFI and PSD using the XGBoost regressor. This confirms the utility of the predicted indices, being highly correlated with the actual ground truth MD.

### Prediction of local indices

#### Pointwise sensitivity values

**Fig. 3:**
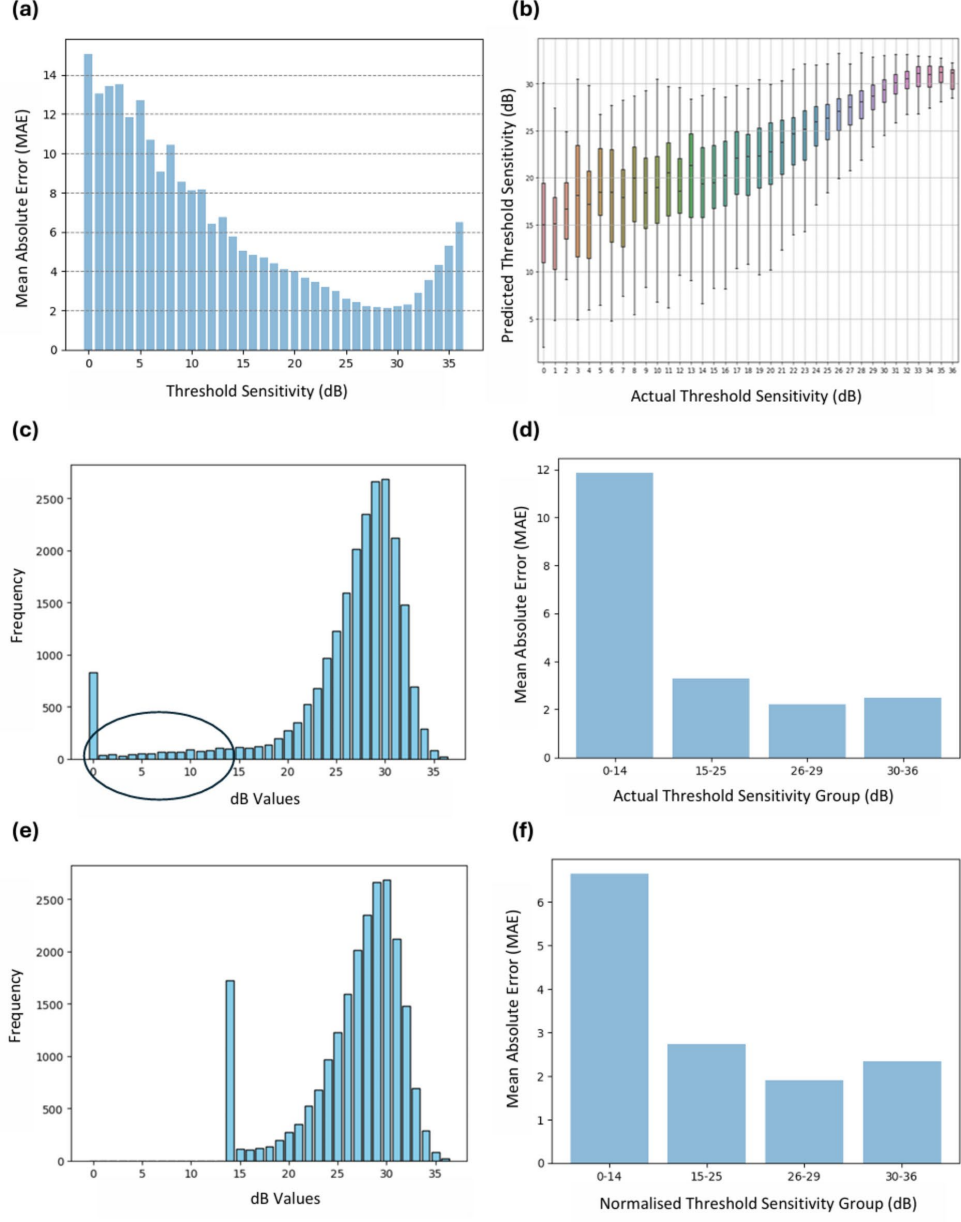
Illustration of (**a**) mean absolute error (dB) vs baseline threshold sensitivity (RF regressor). The blue bars represent the mean value. (**b**) Predicted pointwise threshold sensitivity (dB) vs actual baseline threshold sensitivity values (dB) (RF regressor). The box plots represent the range across the median. (**c**) Counts of threshold sensitivity (dB) values in the original data. (**d**) Stepwise prediction report of original data (RF regressor). (**e**) Counts of threshold sensitivity (dB) values after normalising 0–14 dB to 14 dB. (**f**) Stepwise prediction report after normalising (RF regressor).

The OCT data was also used to predict the 52 local VF threshold sensitivity values, excluding two test grid locations at the blind spots. The MAE and predicted threshold sensitivities (TS) were plotted against the baseline TS values to observe the pointwise prediction performance (Table 4 and Fig. 3). The plot of the number of data points for different dB intervals (Fig. 3a) shows that the number of data points below 20 dB is lower than the number of data points above 20 dB. The plot of MAE (and the ranges) vs actual threshold sensitivity (Fig. 3b) reveals that the predictions below 20 dB show a higher variability. With the original data points, the regressors produce a MAE ranging from 3.23–3.52 dB, with SVM producing the lowest MAE of 3.23 dB (95% CI: 3.19–3.27).

**Table 4:** Local prediction performance of VF threshold sensitivity values from OCT data (including augmented data using MICE and SMOTE).

ML Model	Folds	Threshold Sensitivity (dB)		Grayscale Pixel values	
		MAE (original TS)	MAE (normalised TS)	MAE	MSSI
XGBoost	Fold-1	3.37	2.63	11.35	0.70
	Fold-2	3.37	2.66	12.28	0.69
	Fold-3	3.77	2.95	11.84	0.71
	Fold-4	3.58	2.75	10.62	0.72
	Fold-5	3.51	2.85	10.99	0.70
	**Mean ± STD**	3.52 ± 0.17	2.77 ± 0.13	11.42 ± 0.66	0.70 ± 0.01
	**95% CI**	3.37–3.67	2.65–2.88	10.84–11.99	0.69–0.71
SVM	Fold-1	3.27	2.55	10.33	0.75
	Fold-2	3.26	2.53	10.86	0.74
	Fold-3	3.15	2.45	11.29	0.75
	Fold-4	3.23	2.52	10.44	0.75
	Fold-5	3.26	2.51	9.84	0.75
	**Mean ± STD**	**3.23 ± 0.05**	**2.51 ± 0.04**	**10.55 ± 0.55**	0.75 ± 0.01
	**95% CI**	3.19–3.28	2.48–2.54	10.07–11.04	0.75–0.75
RF	Fold-1	3.23	2.49	10.90	0.78
	Fold-2	3.18	2.50	11.78	0.76
	Fold-3	3.71	2.84	11.36	0.77
	Fold-4	3.34	2.54	10.40	0.78
	Fold-5	3.35	2.71	10.50	0.77
	**Mean ± STD**	3.36 ± 0.21	2.62 ± 0.15	10.99 ± 0.58	**0.77 ± 0.01**
	**95% CI**	3.18–3.55	2.48–2.75	10.48–11.5	0.77–0.78

Boldface numbers indicate the best performance per metric. The OCT data includes augmented data using MICE and SMOTE.

#### Introducing the TS normalisation and step size concept

Considering the lower reliability below 20 dB, the ‘step size’ concept was utilised based on the study by Wall et al.^56^, which was further classified in four steps by Phu et al.^41^. Here, four brackets are used to categorise the dB sensitivity values, i.e., sensitivity < 15 dB, 15–25 dB, 26–29 dB, and ≥ 30 dB^41^. At first, all the original threshold sensitivity values are considered to produce stepwise results (Fig. 3d), which shows a higher MAE for the 0–14 dB group.

To address VF variability, values below the 14 dB sensitivity cut-off were normalised to 14 dB (Fig. 3e) in the target values to stabilise predictions (Fig. 3f). OCT thickness values, although prone to the ‘floor effect’^57^, were retained as they are crucial clinician inputs to the models. This decision was made to preserve subtle structural variations that are critical for predicting VF sensitivity, but also to maintain model interpretability. Normalising the VF sensitivity values less than 15 dB improves the performance of the prediction models, resulting in lower prediction differences (i.e., MAE), particularly in the 0–14 dB group, where the MAE has been reduced substantially compared to models trained on the original (not normalised) TS data (Fig. 3, Table 4). With the TS normalised data points, the regressors show improved performance with a MAE ranging from 2.51–2.77 dB, where SVM produced the lowest MAE of 2.51 dB (95% CI 2.48–2.54).

We compared the performance of TS prediction using non-normalised and normalised TS values against the clinical test–retest variability of pointwise sensitivity reported by Tan et al.^58^ (data points extracted using a custom Python program). The results indicate that the MAE obtained using the normalised data points (trained and tested with RF regressor) is comparable with the clinical test–retest variability, demonstrating improved agreement and reliability^58^ (Supplementary Fig. 3).

Using the original and clean data in training and testing (fivefold CV) without artefacts and oversampling, the regressors show comparatively improved performance in TS prediction, with the lowest MAE of 2.93 dB (RF) without normalising the VF TS, and 2.49 dB (RF) with TS normalisation (Table 5). When a holdout validation was done using the original data points as training and partially augmented data points as the test set, the performance comparatively reduced, with the lowest MAE of 4.07 dB (SVM) with original TS data and 2.99 dB (RF) with the normalised data (Supplementary Table 3). However, in all cases, normalising the TS improved the performance of the regressors.

**Table 5:** Local prediction performance of VF threshold sensitivity values from OCT data (excluding any augmentation using MICE and SMOTE).

ML model	Folds	Threshold sensitivity (dB)		Grayscale pixel values	
		MAE (original TS)	MAE (normalised TS)	MAE	MSSI
XGBoost	Fold-1	3.13	2.60	9.50	0.74
	Fold-2	2.98	2.51	10.77	0.71
	Fold-3	3.39	2.85	11.2	0.71
	Fold-4	3.06	2.65	10.83	0.71
	Fold-5	3.20	2.66	10.84	0.71
	**Mean ± STD**	3.15 ± 0.16	2.66 ± 0.13	10.63 ± 0.65	0.72 ± 0.01
	**95% CI**	3.01–3.29	2.55–2.77	10.06–11.2	0.71–0.72
SVM	Fold-1	3.24	2.82	9.32	0.77
	Fold-2	3.00	2.59	10.31	0.75
	Fold-3	3.34	2.93	11.34	0.75
	Fold-4	3.13	2.76	9.63	0.76
	Fold-5	3.35	2.86	9.87	0.76
	**Mean ± STD**	3.21 ± 0.15	2.79 ± 0.13	**10.09 ± 0.79**	0.76 ± 0.01
	**95% CI**	3.08–3.34	2.68–2.9	9.4–10.78	0.75–0.76
RF	Fold-1	2.84	2.40	9.48	0.80
	Fold-2	2.87	2.41	10.54	0.78
	Fold-3	3.18	2.70	10.68	0.78
	Fold-4	2.77	2.41	10.77	0.77
	Fold-5	2.98	2.54	10.19	0.78
	**Mean ± STD**	**2.93 ± 0.16**	**2.49 ± 0.13**	10.33 ± 0.53	**0.78 ± 0.01**
	**95% CI**	2.79–3.07	2.38–2.61	9.87–10.79	0.77–0.79

Boldface numbers indicate the best performance per metric. The results are obtained from the clean data without MICE and SMOTE-based augmentation.

#### Prediction of grayscale images

**Fig. 4:**
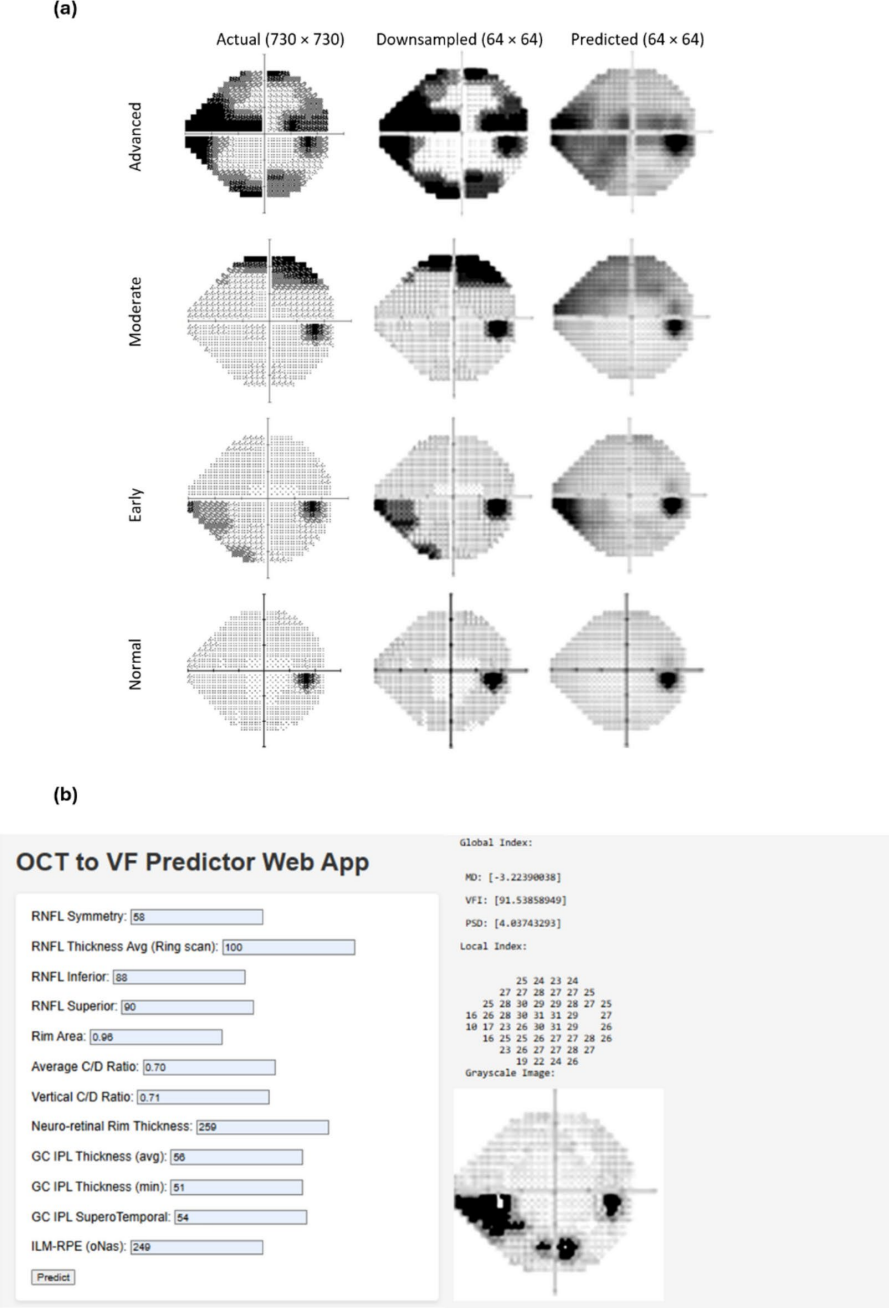
(**a**) Illustration of the prediction of grayscale VF image for different stages of glaucoma and a normal subject. (**b**) A snapshot of the input and output windows of OCT to VF predictor app.

The OCT-based features (RNFL, GC and MC features) were used to predict the grayscale images. This was done by the regressors such that all OCT data is used as input features to predict each of the grayscale pixel intensities (output) to reconstruct the predicted image with all the pixel outputs (Fig. 4a). To do this, at first, the original images (width × height = 730 × 730 pixels) were downsampled (64 × 64 pixels), and used as target outputs for the multi-output regressors. Once the outputs were predicted, those predicted pixel values were used to reconstruct the grayscale image.

The performance of the grayscale image prediction was evaluated by using MAE and MSSI metrics, which consider the texture and structure of the original and predicted images. The results indicate that SVM achieved the lowest MAE of 10.55 (95% CI 10.07–11.04). Considering MSSI, the RF regressor performed best with a MSSI of 0.77 (95% CI 0.77–0.78). The other regressors showed reasonable performance with XGBoost producing the lowest MSSI of 0.70 (95% CI 0.69–0.71) and SVM producing a MSSI of 0.75 (95% CI 0.75–0.75) (Table 4).

Using the original and clean data to train and test (fivefold CV) without artefacts and oversampling, the regressors show comparatively improved performance in grayscale image prediction, with the lowest MAE of 10.09 using the SVM regressor and highest MSSI of 0.78 using the RF regressor (Table 5). When a holdout validation was done using the original data points as training and partially augmented data points as the test set, the performance comparatively reduced, with the lowest MAE of 12.32 dB and highest MSSI of 0.76 using the RF regressor (Supplementary Table 3).

From the qualitative assessment of some example predicted grayscale images (Fig. 4a), the predicted grayscale images for normal and early glaucoma show clear functional vision loss. However, the predicted moderate VF seems significantly different from the actual one—missing the fine-grained details in the superonasal region. This example can be explained by the RNFL/GCIPL thickness feature’s priority by XML, which might contribute to the inaccurate prediction in the VF sectors. Also, this might be due to the fact that OCT parameters have reached the measurement floor^59^, which results in the failure of the regressors model to capture the spatial details in the superonasal region and affects the prediction of the grayscale image.

### Explainable ML results

An XML-based analysis was performed on the RF regressor trained to predict global index MD from OCT data (R = 0.76). The topmost features identified by the SHAP analysis were plotted in a scatter plot and SHAP dependence plot to understand the impact of the features on the prediction. Then a set of selected limited input features was used to predict the pointwise threshold sensitivities again, to observe the effect of using limited features in the pointwise predictions.

#### SHAP feature ranking

**Fig. 5:**
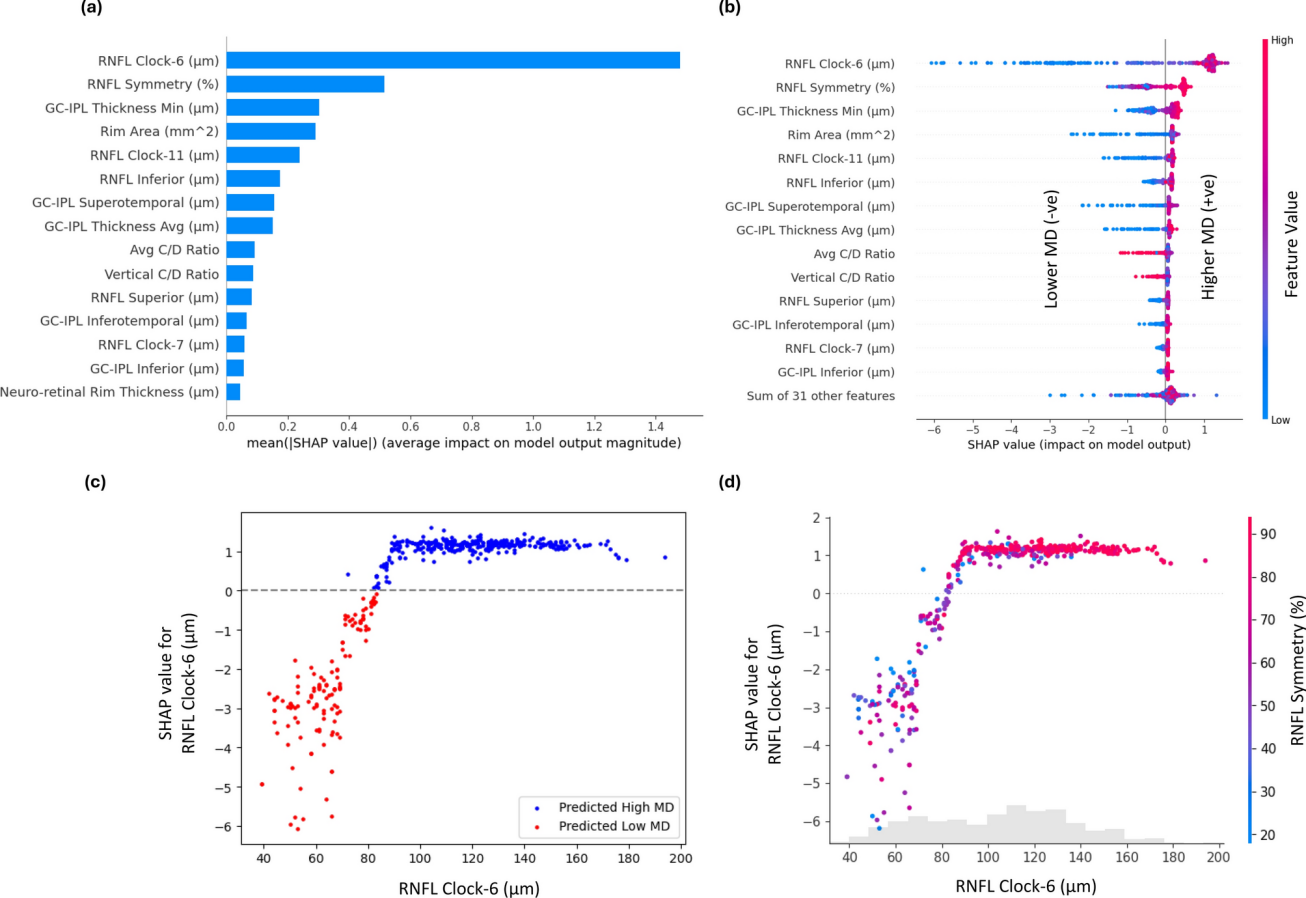
Results of SHAP analysis for VF prediction form OCT using the RF model. (**a**) SHAP feature ranking. (**b**) SHAP swarm summary plot. (**c**) SHAP dependence plot for RNFL Clock Hour-6. (**d**) SHAP feature interaction plots for RNFL Clock Hour-6 and RNFL Symmetry.

Several features were identified as the most important features by SHAP analysis (Fig. 5a). The SHAP feature ranking shows that RNFL clock hour-6 (part of RNFL inferior) is the topmost feature, with Rim area, GC-IPL thickness value (minimum), and RNFL Symmetry, respectively being other top features. The other important features include RNFL thickness at the inferior quadrant and Vertical C/D ratio.

In the SHAP summary plots (Fig. 5b), positive SHAP values on the right side influence the model towards predicting a win, representing a higher probability of predicting higher MD values (or more positive MD values, likely normal subjects) (Fig. 5b), while negative SHAP values on the left steer the model towards predicting a loss, indicating a higher probability of predicting lower MD values (i.e., more negative MD, likely glaucoma) (Fig. 5b). The plot also illustrates that higher rim area, GC-IPL thickness and RNFL symmetry (Fig. 5b) correlate with an increased likelihood of predicting positive MD (likely normal subjects), whereas lower values of these features (Fig. 5b) possess an increased likelihood of predicting lower MD values (or negative MD values, likely glaucoma). Average and vertical C/D ratios show the opposite trend, i.e., smaller values of the C/D ratio correspond to more negative MD values (likely glaucoma) while larger C/D ratio corresponds to more positive MD values (normal).

#### SHAP dependence plots

To understand the contribution of each feature from each data sample to the model prediction corresponding to their magnitude, a SHAP dependence plot was created for the top-ranking OCT features (Fig. 5c, Supplementary Fig. 4 (a–c)). In the plots, positive values on the vertical axis indicate that the feature values positively influence the prediction, while negative values signify a negative impact. Points near the zero line suggest instances where a feature did not significantly contribute to the model’s prediction. The plot confirms that lower RNFL clock hour-6 thickness (Fig. 5c), RNFL symmetry (Supplementary Fig. 4a), GC-IPL thickness (Supplementary Fig. 4b) and Rim area (Supplementary Fig. 4c) correlates with higher likelihood of predicting lower or more negative MD (likely glaucoma), while higher values of these features predict higher or more positive MD (likely normal), with a crossover around the magnitude of 80 μm, 75%, 60 μm and 0.7 respectively.

While RNFL clock hour-6, RNFL symmetry, Rim area, and minimum GC-IPL thickness are the most influential features, the SHAP feature interaction plot of RNFL symmetry with other top-ranking features clarifies its interpretation (Fig. 5d, Supplementary Fig. 4d–f). The feature interaction between RNFL clock hour-6 and RNFL symmetry (Fig. 5d) indicates that lower RNFL clock hour-6 thickness (Fig. 5d) along with lower RNFL symmetry, increases the chance of predicting lower or negative MD (likely glaucoma). Similarly, lower minimum GC-IPL thickness (Supplementary Fig. 4d) or Rim area (Supplementary Fig. 4e), along with lower RNFL symmetry, increase the chance of predicting lower or negative MD (likely glaucoma). In Supplementary Fig. 4f, the lower Rim area combined with lower minimum GC-IPL thickness further increases the likelihood of predicting lower or negative MD (likely glaucoma).

### App development (ML-model deployment as a web app)

Using the explainable results, a web-based tool (OCT to VF Predictor App) was developed for clinicians to assist in glaucoma diagnosis. All the RF-based regression models—three for global indices prediction (MD, PSD and VFI) and two for pointwise sensitivity prediction (pointwise sensitivity values and grayscale images, which are the visual representations of pointwise sensitivities)—were deployed in the same app.

The models were trained using the most important SHAP-based features, allowing clinicians to input these values. Among the top features selected by SHAP analysis, the correlated features were filtered based on the clinician’s recommendation and previous literature, for example, RNFL clock hour-6 and RNFL clock hour-7 are part of RNFL Inferior thickness, so RNFL Inferior thickness was selected as a global level feature. Similarly, RNFL clock hour-11 is a part of RNFL Superior thickness, so RNFL Superior thickness was considered, and finally, 12 features were selected. Developed with the Flask framework and HTML templates, the tool follows a client–server architecture, enabling clinicians to interact with the web application through a web browser, while the server manages requests and responses (Fig. 4b). The web application takes the top OCT features identified by SHAP as input from the clinician and outputs the predicted global VF indices with the individual threshold sensitivity values as a right eye format (excluding blind spots). With the limited number of features (12 features), the RF regressor produced MAE = 2.20 dB, 5.18 (%), 1.58 dB, R = 0.78, 0.77, 0.74, respectively for MD, VFI, and PSD prediction. For the pointwise sensitivities, the RF model produced MAE = 2.73 dB (normalised TS) and 3.47 dB (original TS), and for grayscale image generation, it showed a MSSI of 0.76.

## Discussion

In this study, we have developed a process where VF parameters can be reliably predicted from OCT parameters. The XML analysis revealed that RNFL clock hour-6 (RNFL inferior area), Rim area, and minimum ganglion cell thickness, followed by RNFL symmetry, were the most effective predictors for determining the MD global indices along with 8 other features. These selected 12 features obtained from SHAP analysis demonstrated promise in predicting MD, VFI, and PSD and local threshold sensitivities with mean absolute errors of 2.20 dB, 5.18 (%), 1.58 dB, and 3.47 dB respectively. Employing SHAP provided deeper insights into feature importance and decision boundaries. The study results can be discussed based on the performance of differential regression models, the implications of the findings, study limitations and potential avenues for future research.

Using the OCT spatial domain features with ML models in this study, the achieved performance demonstrates the potential of the proposed approach within the context of our specific dataset (CFEH dataset), both in global predictions (MD, VFI and PSD) and local predictions (52 threshold sensitivities). However, we acknowledge that direct comparisons with existing deep learning-based studies are limited by differences in datasets, input types, and experimental settings. A head-to-head comparison, using the same dataset and evaluation protocol, would be required for a more definitive assessment of relative performance.

While predicting MD using all 45 spatial domain features, we achieved an R-value of 0.76 and MAE of 2.28 dB, which was improved to R = 0.78 and MAE = 2.20 dB respectively using the reduced 12 features and the RF model with SHAP analysis. Our regression model performance on MD prediction using the RF model outperformed that of Mohammadzadeh et al.^22^ (R = 0.74 and MAE = 3.5 dB using 3D-DenseNet121) and Mark et al.^23^ (MAE = 3.7 dB using ResNet50), and Hemelings et al.^24^ (MAE = 2.89 dB using Xception), all of whom used deep learning for the same task. In terms of predicting local threshold sensitivity values, we achieved MAE = 3.23 dB (SVM) (original TS) and 2.51 dB (normalised) using the 45 spatial domain features, which surpass the performance of Mohammadzadeh et al.^22^ (6.5 dB using 3D DenseNet), Zhu et al.^25^ (4.9 dB using a radial basis function customized under a Bayesian framework (BRBF)), Lazaridis et al.^26^ (3.6 dB using a multichannel variational autoencoder [MC-VAE]), Hemelings et al.^24^ (4.82 dB using a customised deep learning (DL) regression model with Xception backbone) and Moon et al.^27^ (3.10–3.17 dB using Inception-ResNet-V2). In our study, when normalising the values below 14 dB in local sensitivity values prediction using 45 spatial domain features, we achieved a MAE of 2.51 dB, which is 0.72 dB lower than with the original TS (3.23 dB) using the SVM regressor. With a reduced number of explainable features, the RF regressor’s performance is also comparable with a MAE of 2.73 dB with floored data and 3.47 dB with original TS. It is important to acknowledge that the deep learning-based studies we compared our results with used datasets from different sources, which may limit the direct comparability of performance metrics. However, the MAE versus actual threshold sensitivity plot from our study (Fig. 3a,d,f) is comparable to the mean test–retest difference versus threshold sensitivity values reported by Tan et al.^58^, who partially used data from the same source as ours (CFEH dataset). This similarity strengthens the relevance of our findings to the existing clinical literature.

There is indeed a lack of consistency of accuracy across the spectrum of glaucoma severity, with predictions being more accurate for normal and early glaucoma cases than for moderate and advanced stages. The overall correlation coefficient we reported is likely influenced more by the normal and early glaucoma cases than by the moderate and advanced cases. The scatter plots for our predictions align with findings from other published works on VF prediction, such as those by Kim et al.^60^. However, the poorer performance in moderate and advanced cases may be attributed to the OCT measurement floor effect^59^, where severe VF loss limits the range of measurable predictions^57,61^. Despite this, some studies focusing on glaucoma diagnosis using OCT data with AI demonstrate higher diagnostic accuracy for moderate and advanced glaucoma compared to early cases. For example, Wu et al.^62^ employed five classifiers to classify glaucoma stages using OCT data, achieving accuracies of 0.69–0.86 for early glaucoma, 0.83–0.93 for moderate glaucoma, and 0.87–0.95 for advanced glaucoma, with an overall accuracy of 0.74–0.88. These findings underscore the importance of accurate VF prediction in early glaucoma cases, as they may contribute significantly to a multimodal diagnostic approach, where OCT-based unimodal methods alone may fall short in early-stage detection. Future studies should consider including glaucoma suspects^63^ in the patient cohort, as this group is critical for early glaucoma diagnosis and could improve the utility of multimodal approaches.

The example of predicted grayscale images shows that they are more accurately predicted in the inferotemporal region; however, in some instances, the regression models could not predict well in the superonasal region of the VF (see the example of moderate glaucoma in Fig. 4a). A possible explanation could be RNFL nasal thickness, RNFL inferonasal or RNFL inferotemporal thickness being the lower rank according to the explainable ML. As mentioned by Mark et al.^23^, when they used deep learning, the prediction of superior and superonasal sectors of the VF relied on the inferior region of ONH scans. Although RNFL inferior thickness appears to be one of the top-ranked features identified by SHAP analysis in our study, the lower priority of the RNFL inferotemporal, RNFL inferonasal or GC-IPL inferotemporal thickness by the regression models might be contributing to less accurate prediction on superonasal region of the VF. Conversely, GC-IPL supertemporal thickness appears to be one of the top features identified by SHAP, which might be contributing towards more accurate prediction in the inferotemporal area (see the example of early glaucoma in Fig. 4a). Another possible explanation could be the “measurement floor” of OCT thickness values, which limits their ability to capture the structure–function relationship in moderate to advanced glaucoma patients^57,61^.

The introduced TS normalisation and step-size concept based on clinical studies addresses both data imbalance and high test–retest variability issues. Combining the TS normalisation and the step-size concept, we achieved a MAE reduction of 5 dB for the 0–14 dB step size. Normalising the sensitivity values from 0–14 dB to 14 dB likely improved the regression model’s performance because the adjustment narrows the range of target values (0–36 dB to 14–36 dB) and mitigates the effects of higher variability and measurement errors in the lower sensitivity range (below 14 dB). All the prediction models developed in this study use ML models with CIRRUS extracted features, which could be combined with an OCT machine, with minimal feature inputs to predict possible maximum VF indices.

The “floor effect” is often associated with OCT measurements—where and further thinning in advanced glaucoma is not captured and thus the changes in the RNFL thickness do not appropriately reflect glaucoma progression^57,59^. However, we chose 14 dB as the cutoff for VF sensitivity to align with clinical interpretations of functional vision and kept the OCT values intact. VF sensitivity below 14 dB often reflects the limitations of psychophysical measurement methods, where the probability of detecting perimetric stimuli becomes unreliable due to response saturation^16^. As a result, TS points in this range cannot be differentiated further, making them clinically insignificant. From the machine learning perspective, normalising OCT inputs could introduce unreliable results by removing subtle structural variations essential for predicting VF sensitivity, impairing the model’s performance. Therefore, normalisng both OCT and normalising VF sensitivity could distort the relationship between structure and function, leading to poor generalisation during inference. Moreover, clinicians rely on real, unprocessed OCT data, and modifying inputs through normalising could reduce trust and interpretability. However, our explainability approach ensures that low-ranked and less-relevant OCT features are excluded—which might be prone to flooring effects, enhancing the model’s reliability and trustworthiness. Future work could explore using other explainable methods, such as Partial Dependency Analysis (PDA)^64^ to identify the OCT flooring effect to determine any cut-offs on the VF predictions but also predict OCT from VF using explainable ML for deeper insights.

The explainable OCT features in this study identified by the SHAP analysis show promising results when compared with studies using deep learning-based features. With a cohort comprising glaucoma and normal, the main task of this study was to predict the VF indices using OCT data. However, explainable analysis applied to the regressors produced some interesting outcomes as a byproduct, which is useful in glaucoma diagnosis and interpretation. The SHAP analysis applied to the MD regressor relates the OCT feature magnitudes with the possibility of predicting high or low MD of VF. According to the given criteria by Mills et al.^37^, a more negative MD value correlates with a higher severity of glaucoma and vice versa. Therefore, a predicted positive MD value from OCT indicates a higher likelihood of the patient being normal, while a predicted low or more negative MD value suggests a higher likelihood of the patient having glaucoma^37^. Also, the top features identified in this study are consistent with contemporary clinical research, which highlights features responsible for glaucoma diagnosis. For example, RNFL clock hour-6 (inferior), rim area, minimum GC-IPL thickness and RNFL symmetry appear to be the most important features for VF prediction from OCT in this study. Han et al.^65^ reported a correlation of R = − 0.75 of rim area and disc damage likelihood scale (DDLS) due to glaucoma, while Hong et al.^66^ reported interocular RNFL thickness symmetry (AUC = 0.96) to be one of the contributors to the diagnostic modality of early glaucoma. Similarly, Wu et al.^62^ reported RNFL inferior to be prominently influential in glaucoma detection, which supports the explainable results using SHAP in our study.

The present study varies from other ML-based studies on glaucoma diagnosis in terms of both feature engineering and VF global and local measure prediction. First, this study uses SHAP analysis, a game theoretical explainable approach to identify the most important features. Second, for VF global and local measure prediction, we leveraged ML for multi-output prediction and achieved better results than existing deep learning-based studies. Deep learning-based models such as convolutional neural networks (CNN) are computationally demanding and require high-end graphical processing units (GPUs) for training. On the other hand, the ML models used in this study are computationally less expensive and can even be trained on central processing units (CPUs). Also, the developed app can be used on portable devices, such as mobile phones.

Besides the strengths, it is also worth mentioning the limitations and scope. First, while predicting local sensitivity values, this study shows high variability in prediction below 20 dB (i.e., 19 dB) similar to other studies^58^. This could be due to (i) the measurement floor effect below 20 dB^67^ and (ii) limited training data below 20 dB compared to the other data points. As Gardiner et al.^16^ state, sensitivity measurements are more variable when pointwise sensitivities are below approximately 15 to 19 dB because of a reduction in the asymptotic maximum response probability. Lee et al.^68^ have commented on the limited training data below 20 dB, which could be one of the reasons behind higher variability.

Second, we utilised the MICE algorithm to impute missing OCT features in some cases and applied SMOTE-based oversampling to balance the minority class (normal subjects). When the test set included only augmented data with a holdout validation approach, the performance was comparatively lower than the results obtained from the original data points. However, MAE was within the 24-2 Humphrey Field Analyzer (HFA) standard global test–retest variability^55^. Thus, the prediction performance demonstrated robustness to OCT artefacts associated with the 5.2% missing samples. Although this is a regression problem, we used a smaller sample size and the imbalance ratio between normal subjects and glaucoma cases (0.84) indicates that the dataset was not significantly imbalanced, and the performance metrics with and without the augmented data were relatively consistent. Using a large dataset and exploring alternative imputation techniques such as k-Nearest Neighbour (k-NN) imputation and oversampling techniques such as Adaptive Synthetic Sampling (ADASYN) could be considered as possible future work.

Third, we acknowledge the lack of external testing is a limitation of this study, as the performance of the proposed model has only been validated on the internal dataset (collected using CIRRUS HD-OCT). Therefore, the generalisability of the regression models to broader populations or different clinical settings (e.g., OCT machines) remains to be demonstrated. Relating to a single centre (CFEH), glaucoma suspects and those with glaucoma presenting at the centre have been extensively used in past publications as they reflect disease entities within a multicultural society^4,5,36,69–71^. Future work will aim to include external datasets including other data from other OCT machines for testing to ensure the robustness and broader applicability of the model across diverse scenarios. Lastly, our study utilised only OCT spatial domain features for the VF prediction. Various frequency domain features could be derived from the double-hump pattern of 256 temporal-superior-nasal-inferior-temporal (TSNIT) patterns, such as the power spectral density of the frequency domain transformed pattern, which might improve the performance. The use of multimodal data, such as colour fundus images and other clinical information such as age, gender, and other medical records could also help improve prediction performance. In that case, adding privacy-preserving mechanisms to the regression models would add value to preserve privacy of the sensitive medical data, which might facilitate the incorporation of the app in the future Internet of Things (IoT)^72^. These options could be considered in future studies.

## Supplementary Information

Supplementary Information.

## Data Availability

The raw data are not available for public access, but the statistically analysed OCT data in tabular format for a group of patient cohorts (normal, glaucoma/glaucoma stages) may be accessible upon reasonable request to the corresponding author.
