# Supplementary material for: Predicting visual field global and local parameters from OCT measurements using explainable machine learning
Source: Sci Rep. 2025 Feb 16;15:5685. doi: 10.1038/s41598-025-89557-1 (PMC11830782; doi:10.1038/s41598-025-89557-1)
Supplement: Supplementary file 1 — Supplementary Information. [file 41598_2025_89557_MOESM1_ESM.pdf]

Supplementary Information

Predicting visual field global and local parameters from OCT measurements using explainable machine learning

Md Mahmudul Hasan[a,*], Jack Phu[b,c,d,e], Henrietta Wang[b,c,e], Arcot Sowmya[a], Erik Meijering[a], Michael Kalloniatis[b,e,f]

[a] School of Computer Science and Engineering, University of New South Wales, Sydney, NSW, Australia
[b] School of Optometry and Vision Science, University of New South Wales, Sydney, NSW, Australia
[c] Centre for Eye Health, University of New South Wales, Sydney, NSW, Australia
[d] Faculty of Medicine and Health, University of Sydney, Camperdown, NSW, Australia
[e] School of Medicine (Optometry), Deakin University, Waurn Ponds, VIC, Australia
[f] University of Houston College of Optometry, University of Houston, Houston, TX, United States of America

[*] *Corresponding author*
Md Mahmudul Hasan
School of Computer Science and Engineering
University of New South Wales, Sydney, NSW 2052, Australia
Email: md_mahmudul.hasan@unsw.edu.au

Short title: Predicting visual fields from OCT measurements using explainable ML

Keywords: 24-2 test grid, optical coherence tomography, glaucoma, explainable machine learning, SHAP analysis, perimetry, visual fields

Supplementary Tables

Supplementary Table 1: Spatial domain features extracted using CIRRUS HD OCT software for VF prediction from OCT.

RNFL Thickness Analysis	GC-IPL Thickness Analysis	Macular Thickness Analysis
RNFL symmetry (%)	Average GC-IPL thickness	ILM-RPE thickness-central subfield
Average RNFL thickness (µm)	Minimum GC-IPL thickness (µm)	ILM-RPE thickness-volumetric cube
RNFL thickness ring area (mean) (µm)	GC-IPL superior (µm)	ILM-RPE thickness-average cube
RNFL thickness in quadrants (µm) (count=4)	GC-IPL superonasal (µm)	ILM-RPE centre-foveal
• RNFL superior	GC-IPL inferonasal (µm)	ILM-RPE inner superior (iSup)
• RNFL nasal	GC-IPL inferior (µm)	ILM-RPE inner nasal (iNas)
• RNFL inferior	GC-IPL inferotemporal (µm)	ILM-RPE inner inferior (iInf)
• RNFL tempora	GC-IPL-superotemporal (µm)	ILM-RPE inner temporal (iTem)
RNFL clock hours		ILM-RPE outer superior (oSup)
RNFL clock hour 1- 12 (count= 12)		ILM-RPE outer nasal (oNas)
Rim area		ILM-RPE outer inferior (oInf)
Disc area		ILM-RPE outer temporal (oTem)
Average cup to disc ratio		
Vertical cup to disc ratio		
Cup volume		
Neuro-retinal Rim Thickness		
Count (individual analysis) =25	8	12
Total Count= 45		

Supplementary Table 2: Performance for the three different regression models for VF prediction from OCT data with holdout validation, the training set being the clean data and the test set containing the augmented data with MICE and SMOTE. Boldface numbers indicate best performance per metric.

ML Model	MD			VFI			PSD		
	R	RMSE (dB)	MAE (dB)	R	RMSE	MAE	R	RMSE (dB)	MAE (dB)
XGBoost	0.71	3.83	3.02	0.74	9.15	**6.09**	0.67	2.91	1.97
SVM	0.73	3.96	2.80	0.68	12.73	6.45	0.72	3.17	2.21
RF	**0.74**	**3.91**	**2.51**	**0.78**	**8.22**	6.32	**0.82**	**1.65**	**1.16**

Supplementary Table 3: Local prediction performance of VF threshold sensitivity values with holdout validation, the training set being the clean data and the test set containing the augmented data with MICE and SMOTE. Boldface numbers indicate best performance per metric.

ML Model	Threshold Sensitivity (dB)		Grayscale Pixel values	
	MAE (Original TS)	MAE (Normalised TS)	MAE	MSSI
XGBoost	4.88	3.28	12.92	0.68
SVM	**4.07**	3.58	14.09	0.72
RF	4.41	**2.99**	**12.32**	**0.76**

Supplementary Figures

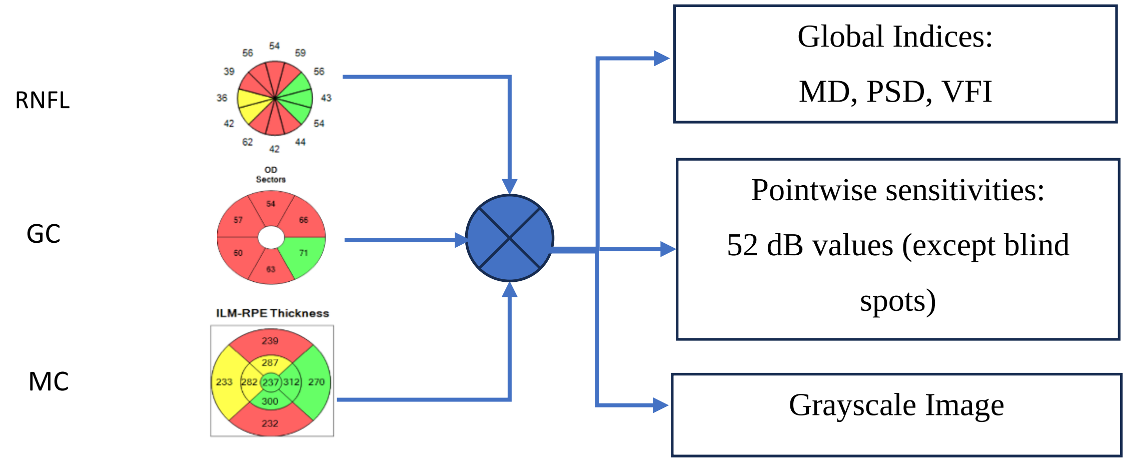

Supplementary Figure 1: Flow chart illustrating the process to predict VF from OCT data.

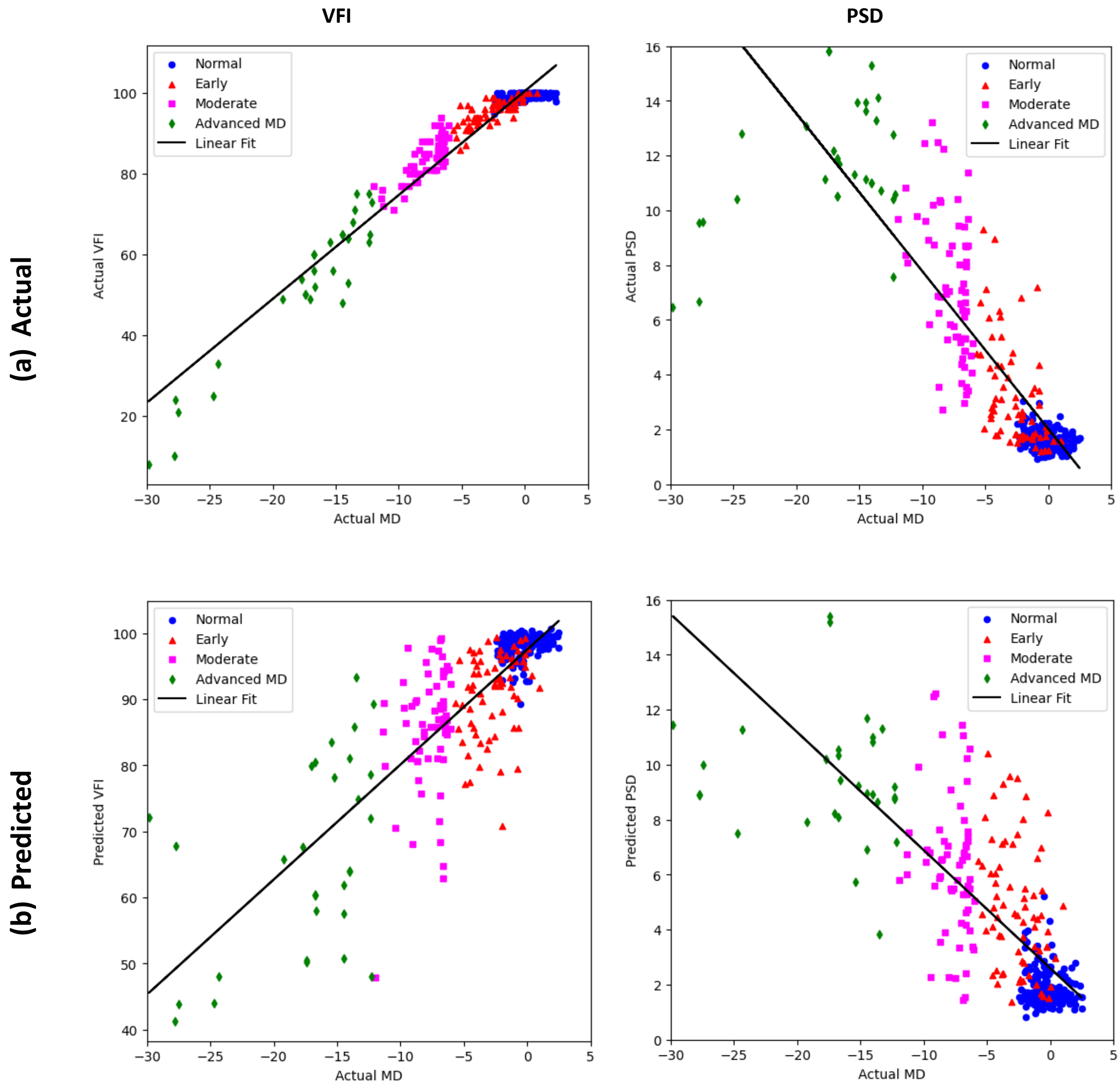

Supplementary Figure 2: Scatter plots of actual and predicted VFI and PSD with Actual MD (ground truth). (a) Actual VFI vs Actaul MD (R= 0.96 using XGBoost) and Actual PSD vs Actual MD (R= -0.83, XGBoost) (b) Predicted VFI vs Actual MD (R =0.80, XGBoost) and Predicted PSD vs Actual MD (R= -0.73, XGBoost). MD: mean deviation, VFI: visual field index, PSD: pattern standard deviation.

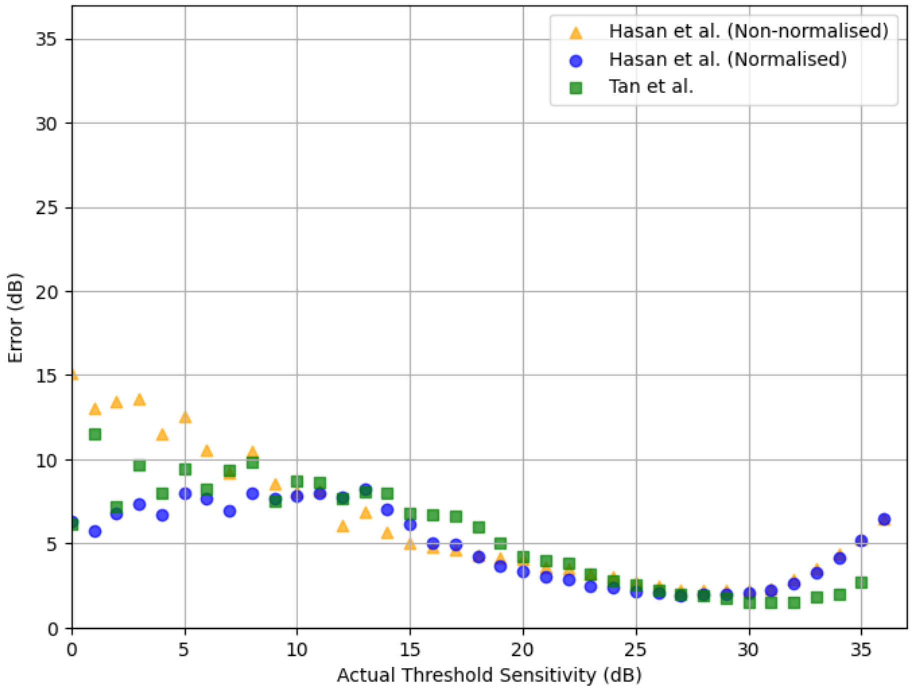

(a)

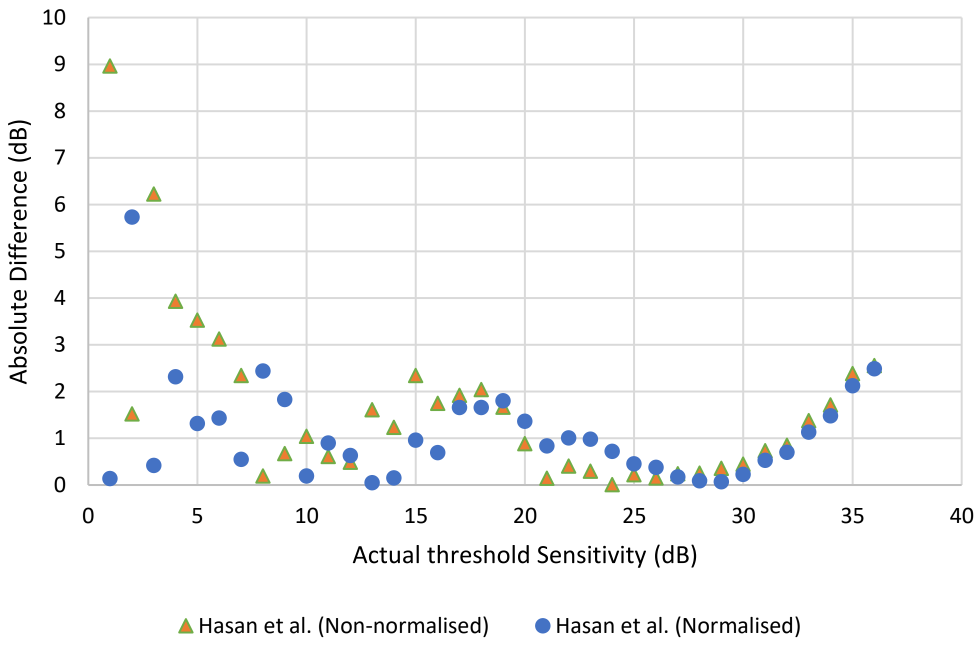

(b)

Supplementary Figure 3: Comparison of TS prediction performance of RF regressor using non-normalised and normalised TS values with the clinical test-retest variability of pointwise sensitivity reported by Tan et al. [1] (a) Error versus Actual threshold Sensitivity; Error in the y-axis indicates the mean absolute error (MAE) for Hasan et al., and test-retest variability for Tan et al. [1] (b) Difference plot of the MAE obtained from Hasan et al. (non-normalised and normalised TS-based results) with the test-retest variability obtained from Tan et al. [1]

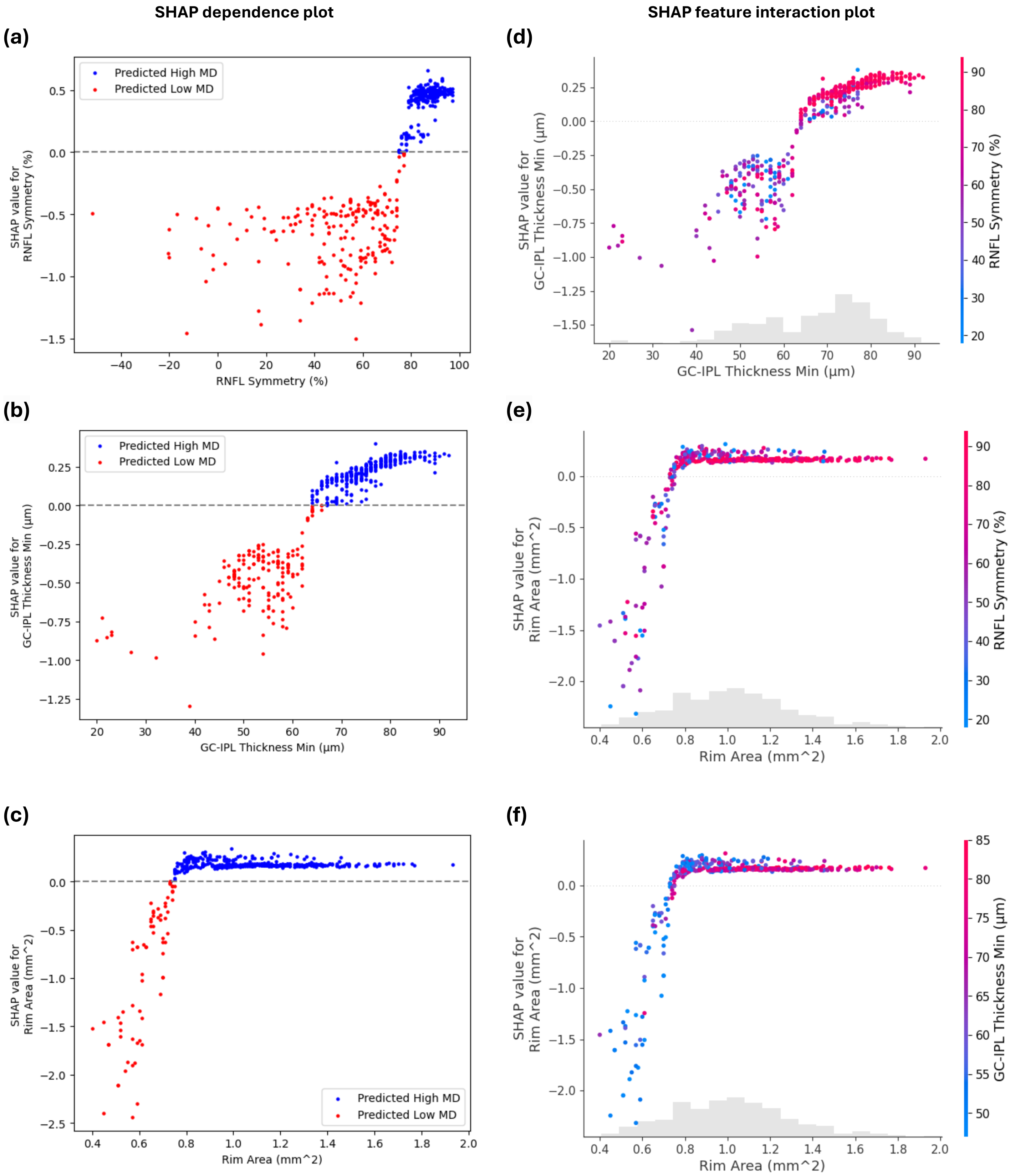

Supplementary Figure 4: SHAP dependence and interaction plots. SHAP dependence plots for (a) RNFL Symmetry (%) (b) Minimum GC-IPL thickness (µm) (c) Rim Area (mm^2). SHAP Feature interaction plots for (d) Minimum GC-IPL Thickness (µm) and RNFL

Symmetry (%) (e)) Rim Area (mm^2) and RNFL Symmetry (%) (f) Rim Area (mm^2) and Minimum GC-IPL Thickness (μm).

Reference

1 Tan JC, Agar A, Kalloniatis M et al. Quantification and predictors of visual field variability in healthy, suspect and glaucomatous eyes using SITA-Faster. *Ophthalmology* 2023.
